# Internet use and pro-environmental behavior: Evidence from China

**DOI:** 10.1371/journal.pone.0262644

**Published:** 2022-01-27

**Authors:** Youzhi Xiao, Xuemin Liu, Ting Ren

**Affiliations:** HSBC Business School, Peking University, Shenzhen, Guangdong, China; Institute for Advanced Sustainability Studies, GERMANY

## Abstract

Solving environmental problems relies upon cultivating pro-environmental behavior in the society. While the internet has been widely used to facilitate information transmission and communication, it’s important to understand its function in promoting pro-environmental behavior. Using the data from the China General Social Survey 2013, the relationship between the use of internet and the individual’s pro-environmental behavior is investigated, and overall positive effects are found. The results show that, the influence of internet use is more pronounced on the private pro-environmental behavior when further dividing pro-environmental behavior into private and public types. Moreover, the positive effect of internet use on pro-environmental behavior is more pronounced among low-income and female groups, compared to middle to high income and male counterparts, respectively, when considering the heterogeneity across different groups of individuals. We further explore the plausible channels of providing information, encouraging participation in pro-environmental campaigns and improving social relationships through which internet use facilitates pro-environmental behavior.

## Introduction

Various environmental problems such as global warming, water shortage and noise have posed threats to environmental sustainability [1]. Since these problems are largely rooted in human behaviors [2, 3], it is essential to understand how to influence and change human behaviors toward environmental issues [4, 5], especially in the current internet era. In general, the pro-environmental behavior refers to the kind of behavior that benefits the environment or at least not harms the environment [6]. Factors such as information, knowledge, education, social relations, social support, social participation and motivation all play important roles in encouraging pro-environmental behavior [7–11].

On the one hand, the internet has developed rapidly in China. According to the China Internet Information Center (CNNIC), which is the state agency accounting for the management and service of the internet under the supervision of the State Internet Information Office, until March of 2020, the internet penetration rate has become 64.5% with 904 million internet users out of the 1.4 billion population in China, significantly higher than the 9.5% global average. Besides, the more recent trend of mobile internet use has also developed rapidly with 454 million users nationwide. With such a rapid popularization and extensive application of the internet, individuals’ lifestyles have also changed substantially. Meantime, the pollution issue has become one of the most severe and urgent challenges for China [12]. The Chinese government has been paying more attention to improving and changing the current environmental situation. In 2016, *the 13th Five-Year Plan for Eco-Environmental Protection* was issued by the State Council, which emphasized the objective of constructing an ecologically friendly society among the key national development strategies during the 13th five-year plan period. Broad and effective participation of individuals are called upon to promote pro-environmental behavior and help solve the environmental issues [13, 14].

With the rapid development of the internet and urgent demand of solving current environmental problems, the combination of the internet and environment has become one of the most prominent issues in China. However, due to the data restriction, the relationship between the internet use and pro-environmental behavior remains unresolved. Considering the use of the internet significantly influences information and knowledge acquisition, individual attitudes, social relations and social participation [15–18], it is meaningful to examine the effect of using the internet on the individual’s pro-environmental behavior. For example, in 2017, the Ministry of Environmental Protection and Tencent, one of the most prominent internet companies in China, formed a strategic cooperation to provide the most authoritative air quality index for the public, which relies on cloud computing, big data technology and Tencent’s city services platform. Also, Ant Forest, a direct payment mobile application developed by Alibaba, another most prominent internet company in China, has enabled users to reduce carbon emission by encouraging people to walk and use public transportation. These examples have shown that the opportunity to study the relationship between the internet use and pro-environmental behavior has emerged under the circumstance of rapid development of the internet and urgent demand of solving current environmental problems in China.

The purpose of this paper is to investigate the relationship between the internet use and pro-environmental behavior. Compared with the extant literature, the contributions are mainly as follows: firstly, we attempt to investigate the role of the internet use on encouraging the pro-environmental behavior, which is an extension of the literature on the factors affecting pro-environmental behavior. Secondly, we further divide the pro-environmental behavior into private pro-environmental behavior and public pro-environmental behavior [19, 20]. The internet use has different influence on these two types of pro-environmental behavior. Thirdly, the heterogeneity of the impact of the internet use on pro-environmental behavior is invested, that is to say, by subdividing the samples by gender, income and location. In order to get robust results, several ways of robustness check are conducted including changing the independent variables, using alternative models and instrument variables to alleviate the concern of endogeneity. Lastly, the plausible channels are explored including providing information, encouraging participation in pro-environmental campaigns and improving social relationships.

We organize the remainder of the paper as follows: Section 2 provides theory and develops hypotheses; Section 3 introduces data, variables and empirical strategy; Section 4 reveals results and robustness checks; Section 5 shows discussions; Section 6 presents conclusions.

## Theory and hypotheses

### Theoretical foundation

There are usually two perspectives in perception of environmental problems: the understanding of various environmental problems, and the awareness of daily behaviors that may pollute environment. Environmental behavior is about all activities taken by people towards resources and environment [21], which have positive or negative influence on environment. Among these behaviors, those with positive effects on environment are called pro-environmental behavior [22]. How to promote pro-environmental behavior through internet use has received increasing attentions. Existing research involves a series of theories in behavior, psychology and cognition, such as the theory of planned behavior (TPB) [23], value-belief-norm theory [24, 25], dual concern theory and attachment theory [25], norm-activation theory [26], as well as social cognitive theory [27] have made outstanding contributions to the analysis of pro-environmental behavior.

Particularly, the theory of planned behavior, proposed by Ajzen [28], is widely applied to explain pro-environmental behavior and its underlying driving factors. TPB believed that human behavior is the result of a well-thought-out plan, which can help individuals understand how to change their behavior patterns. For examples, Astrid et al. [29] used TPB to identify key beliefs underlying pro-environmental behavior in high-school students. Han [24] converged value-belief-norm theory and TPB to explore travelers’ pro-environmental behavior in a green lodging context. As discussed by Yuriev et al. [30], TPB allows researchers to identify the determinants of environmental behavior and subsequently target these factors in interventions. Therefore, TPB can be introduced to support and explain the relationship between internet use and pro-environmental. As an important component of information and communication technology (ICT), the internet has been closely intertwined with human activities over the past decade, also having a far-reaching impact on pro-environmental behavior [15]. Because the internet use leads to non-negligible impacts on individuals’ environmental protection behavior through the five core dimensions of TPB, that is, attitude, subjective norm, perceived behavioral control, behavior intention, and behavior.

Specifically, attitude is related to the positive or negative feelings of an individual. Extant literature has found that environmental attitude, willingness to pay for the environment, and environmental risk perception can promote individuals’ pro-environmental behavior [31]. Zhang et al. [32] finds that in China, internet use makes people significantly less satisfied with the environmental quality and their living conditions. The change of attitude towards a more efficient, green and environmentally friendly lifestyle is urgent [33]. The sharing economy and mobile payment are just typical examples, which have greatly improved personal resource utilization and reduced the carbon emissions in recent years [34]. The four categories of sharing economy including re-circulation of commodities, increasing utilization of durable assets, exchange of services, and sharing of productive assets, lead to constructing sustainable societies through a reduction in the total resources required and reducing the pollutants, emissions and carbon footprints [35]. Subjective norm, refers to the social pressure that an individual feels about whether to take a particular behavior. Obviously, exposure to the internet and pressure from public opinion have given a great deterrent effect to those who are environmentally unfriendly. In addition, the magnitude of impact determined by the strength of salient individuals or groups hidden behind the internet. Regarding perceived behavioral control, it emphasizes when individuals think that the more resources and opportunities they have, the stronger their perceptual behavior control. The amount of information in the internet is huge and can be spread rapidly, by which people can have a good knowledge of the environmental issues, environment-protecting activities, recent environment-protecting news and ways to behave environmental-friendly, that is to say, their environmental knowledge may be improved by the information from the internet. For example, the information about the global warming from the internet makes the individuals understand the harm and solutions to global warming, which have positive direct effects on three types of pro-environmental behavior, including accommodating, promotional and proactive behavior [36]. Behavior intention, reflects the judgment of an individual’s subjective probability of taking a specific behavior. Gong et al. [18] stressed that internet use can enhance residents’ health awareness, thereby indirectly increasing their willingness to conduct pro-environmental behavior. Finally, behavior in TPB can be understood as environmental behaviors actually taken by individuals through the internet use, such as information acquisition, organizing environmental activities, and improving social relations. As a major media, the internet plays a considerable role in providing environmental protection information and have an effect on individual behavior, due to the fact that it can facilitate the speed, cost-effectiveness, interactivity, freedom of expression and global reach for environmental organizations [37].

### Factors that affect pro-environmental behavior

The factors that affect pro-environmental behavior can be divided into external factors and individual drivers [7].

#### External factors

The external factors include economy situation, social norms, convenience, voice prompt intervention, municipal recycling programs, recycling refunds or rebates, holding duration, and the advent of innovative technology [7, 38, 39]. Some literature focuses on the direct influence of external factors exert on the pro-environmental behavior. Yang and Weber [40] revealed individual perceived responsibility of pro-environmental behavior differs by GDP per capita. Zhang et al. [39] believe that a recycle promotion policy can be taken as a handy approach to adjust residents’ recycling behavior and cut through the vicious mixed-disposal. Carkoglu and Kentmen-Cin [41] explore the relationship economic development and pro-environmental behavior based on international Social Survey Program 2010 data, and reveal that people in less developed countries (LDCs) are less supportive of international agreements forcing their country to take necessary environmental measures than are citizens in the developed world, in other words, economy development positively affects the pro-environmental behavior. Besides that, some literature mainly focus on spill-over effects of the external factors, such as environmental publicity, education, tax policies and regulations, which not only influence a certain behavior but many other behavior may also be affected [42–45].

#### Individual drivers

The individual factors include demographic variables and psychological variables [7]. For the demographic variables, literature focus on socio-demographic factors, such as gender, age, education, marital status, place of residence, and personal economic situation [7, 9, 46, 47]. For example, Li et al. [7] and Dupont [48] find that married people may pay more attention to environmental problems than singles and prefer to having more pro-environmental behavior because they may care more about the next generation’s future environmental conditions. Ling and Xu [49] find that residents in wealthier areas tend to have more economic freedom and external opportunities to pursue pro-environmental goals. As for the psychological variables, scholars mainly pay attention to the influence of attitudes, moral, beliefs, related knowledge, awareness, values, social capital and emotions on pro-environmental behavior [10, 50]. For example, Ertz et al. [51] verify that pro-environmental business managers may not be able to influence objective contextual factors that consumers face, but they can improve their pro-environmental behaviors by impacting perceptions, attitudes and concerns.

### Internet use and pro-environmental behavior

The internet has gradually become a type of new driving force for economic growth, which has attracted more attention. The internet has also become an indispensable part in our daily life. An increasing number of literature focuses on the relationship between the internet use and individuals’ daily life including consumption behavior and access to information [52, 53]. As for the influence of the internet use on pro-environmental behavior, we can understand the roles of the internet use from two perspectives.

From an external perspective, the internet, as an innovative technology, can improve the deeper integration of the internet into traditional manufacturing industries, thereby promoting new products, new formats and new business models and modern manufacturing systems [54]. The sharing economy is one of new business models through utilizing the internet [55]. It is defined as an economic form in which the assets or services are shared between private individuals, typically on the internet such as Uber or Airbnb [56, 57]. Li et al. [7] find that with the development and advent of innovative technology, the pro-environmental behavior can be promoted. The internet industry, generally speaking, is one of the innovative technologies and environmental-friendly industries. Specifically, the internet makes the traditional industries more environmental-friendly by resource integration, information sharing, operation optimization, supply chain coordination, demand matching and service value-added via big data, cloud computing and the internet. When traditional industries become more environmental-friendly, on the one hand, more environmental-friendly commodities such as energy-saving products may be put into market, which make the individuals have more opportunities to purchase environmental-friendly commodities and services. On the other hand, when traditional industries become more environmental-friendly, the consumer environmental awareness can also be improved [58], which results in having more pro-environmental behavior. Therefore, we suppose that with the development of the internet, the individuals can use the internet more frequently and their pro-environmental behavior may be improved as well.

Considering the internal level, especially from the perspective of psychological variables, the internet use has effects on acquiring information, encouraging to participating in campaigns and improving social relationships.

Regarding information acquisition. Some studies have found that individuals’ behavior is affected by the information acquired, such as consumption behavior and investment behavior [59]. The internet can be treated as one of the media and the extant literature has found that using media significantly influences the pro-environmental behavior due to various information about environment acquired from medias [60]. Moreover, the internet provides a quick and easy access to specific environmental information [61]. The effective and convenient access to information may lead to better knowledge of environmental issues and cultivate environmental awareness, which may promote the pro-environmental behavior. For example, the information acquired from the internet may change the individuals’ or families’ consumption behavior [37], which is an important part of pro-environmental behavior. Besides, the information about the environmental quality can also change the individual behavior and make them behave more environmental-friendly [32]. Furthermore the information acquired from the internet can increase the individuals’ environmental knowledge, which may positively affect the pro-environmental behavior [62–65]. Therefore, we suppose that using the internet can also increase the environmental knowledge and then their pro-environmental behavior may be affected.

As for participation, the internet can be used as a tool for organizing environmental activities, due to the fact that it can facilitate the speed, cost-effectiveness, interactivity, freedom of expression and global reach for environmental organizations [66]. Through the internet platform and technology, environmental monitoring can be done by the internet, and realize the intellectualization of environmental management, improve the early warning ability of environmental pollution emergencies, and facilitate the public’s in-depth participation in environmental supervision [67]. Furthermore, the internet can be used as a social media to improve social relations, which consequently promotes the pro-environmental behaviors [68]. Han and Xu [69] find that interpersonal communication can influence pro-environmental behavior through affecting environmental risk perception, while social media mainly affects pro-environmental behavior by strengthening the effects of interpersonal communication. In general, using the internet encourages non-political and political public participation such as voting for elections or participating in hearings [70]. Pro-environmental behavior, as one of the public participation, is significantly affected by the internet. To be specific, the non-political pro-environmental behavior include taking part in environmental activities organized by NGO; the political pro-environmental behavior indicates that the internet improves the information transparency of government, by which individuals can take part in decisions about the environment and supervise the environmental protection implemented by the government more effectively. More wildly participation makes people more willing to corporate with government to cope with environment problems [71]. Therefore, we suppose that using the internet encourage people to participate in environmental campaigns and then their pro-environmental behavior may be affected.

Considering improving social relationships, the internet can be used as a social media, by which people can contract and share information. Some literature has supported that using the internet positively increases the social relations because people’s interaction online supplements their face-to-face or telephone communication and increases their participation in voluntary organizations and politics [69]. While Videras et al. [72] have found that social relationships positively influence the pro-environmental behavior because individuals can have access to information and resources through their networks, as well as the social norms they follow. Moreover, the four dimensions of the social relationships, that is, social trust, institutional trust, social networks, and compliance with social norms, make the citizens reduce waste as an index of social relationships when spending leisure time, thus engaging in pro-environmental behavior [73]. Specially, as for NGOs, with the development of the internet, they experience significantly lower internal and external communication costs [74], which make them much easier to organize campaigns. Individuals can participate in such campaigns and establish the social relationships more easily, then the pro-environmental behavior may be increased. Therefore, when taking into account the internet use, social relationships and pro-environmental behavior, we suppose that the internet use improves the pro-environmental behavior.

In summary, whether from an external level or an individual perspective, internet use exerts substantive influence on individuals’ behavior. Thus, we propose Hypothesis 1 that the internet use can improve the pro-environmental behavior.

**H1**: Internet use significantly increases the individual’s pro-environmental behavior.

In addition, the pro-environmental behavior can be classified into public pro-environmental behavior and private pro-environmental behavior [11, 20]. Private pro-environmental behavior refers to the behavior without interacting with others and can be implemented through their own efforts (time and energy) [7], which is more about daily pro-environmental habits such as consumption behavior, maintenance and use of household equipment and waste disposal behavior [75]. The public pro-environmental behavior refers to the behavior with interacting with others or under the policy support and is always performed by individuals as responsible environmental citizenship such as active involvement in environmental groups and demonstrations [7]. On the one hand, the internet use is one of the individual-level behaviors and people are inclined to use the internet for the purpose of private or personal affairs, which may have more effects on private pro-environmental behavior. For example, people would like to search the information about the commodities via the internet actively which may lead to purchasing more environmental-friendly commodities, as one of the private pro-environmental behaviors [76]. The information about green efforts of the hotels posted by the clients in the social media via the internet makes others be more likely to choose the environmental-friendly hotels [64, 77], another private pro-environmental behavior. On the other hand, compared with public pro-environmental behavior, private pro-environmental behavior is more easily to be implemented [51]. Public pro-environmental behavior has to be carried out with the participation and assistance of some organizations. For example, individuals can easily use reusable grocery bags, while participating in environmental citizenship activities such as tree-planting and demonstrations may be limited by available opportunities. The extant literature has found that it is a common phenomenon for low rates of participation in environmental citizenship behavior, as one of public pro-environmental behaviors [78]. The less inclination of engaging in public pro-environmental behavior is mainly because the individuals tend to consider private pro-environmental behavior with more importance and less cost [51]. Therefore, we suppose that the internet use may have more impact on private pro-environmental behavior compared with public pro-environmental behavior. Hypothesis 2 is proposed as follows:

**H2**: Compared with public pro-environmental behavior, internet use has more pronounced impact on private pro-environmental behavior.

### The heterogeneous effects of the internet use on pro-environmental behavior

The extant literature has found that demographic variables which belong to individual factors such as age, social class, place of residence, political status, gender, and income significantly influence pro-environmental behavior [7]. Hence, the heterogeneous influence of the internet use on pro-environmental behavior among different groups should be considered. Specifically, as for income, with income increasing, individuals may have higher requirement for better environment and less environmental damage, therefore, they tend to behave more environmental-friendly [79]. However, high-income and middle-income people have fewer environmental problems in their communities than do their poor counterparts [80]. With the more likely to be exposed to environmental problems, the low-income residents may have more concerns on environmental issues. Hence, there may be a balance between income and pro-environmental behavior. What is more, from perspective of information acquisition, compared with middle-income and high-income individuals, low-income individuals have fewer ways to acquire information and may be more sensitive to the information; hence, they may be more likely to be affected by the information provided by the internet [81]. Therefore, we propose that the pro-environmental behavior among low-income residents may be affected more pronounced compared with others. Hypothesis 3a is proposed as follows:

**H3a**: Internet use has more pronounced influence on individuals’ pro-environmental behavior among low income group compared to middle to high income counterparts.

As for gender, among males and females, the influence may be different. Researchers have found that females show relatively stronger environmental concern and exhibit more environmental friendly than males [82–84]. Stronger concerns on environmental issues indicate that females may focus on more news, information and knowledge about the environment. Exhibiting more environmental friendliness indicates that females may prefer to participate in campaigns on environmental issues while using the internet. Therefore, we suppose that for female, the internet use provides more information about the environment and encourage them to participate in environmental campaigns, that is to say, compared with males, the internet use may have more pronounced influence among females. Hypothesis 3b is proposed as follows:

**H3b**: Internet use has more pronounced influence on individuals’ pro-environmental behavior among females compared to males.

When considering the difference between the urban and rural areas, the residents living in rural areas experience the environment in very different ways from their urban counterparts due to the rural residents are in touch more with nature [80]. We can understand the difference of the pro-environmental behavior between the urban and rural areas from two perspectives. On the one hand, from perspective of the external factors mentioned above. The infrastructure construction and economy development are significantly different between urban and rural areas [85], which may lead to different pro-environmental behaviors between urban and rural residents. On the other hand, from perspective of individual factors, urban residents exhibit greater pro-environmental attitudes and behavior than those living in rural areas due to urban residents are more exposed to environmental degradation and are more likely to be better informed about the issue [86, 87]. In addition, compared with rural residents, it is easier for urban residents to take part in environment-protecting campaigns and purchase environmental-friendly commodities such as China Environmental Label and Green Food labeling products [88]. Therefore, we propose that internet use has more pronounced influence among urban residents. Hypothesis 3c is proposed as follows:

**H3c**: Internet use has more pronounced influence on individuals’ pro-environmental behavior among urban residents compared to rural counterparts.

## Methods

### Sample

We use the Chinese General Social Survey (CGSS) conducted in the year of 2013 for the analysis. The CGSS, as one of a national, comprehensive and continuous Chinese survey projects which was initiated in 2003, conducts a cross-sectional survey of more than 10,000 households among provinces in mainland China each year, However, only the CGSS (2013) has detailed information on respondents’ environmental protection behaviors and attitudes, as well as the frequency of the internet use. CGSS (2013) dataset includes 11,438 individuals. After deleting the sample with omitting information, the remaining sample includes 9,752 individuals. Besides, some macro-economy data is used, which are from China Statistical Yearbook and Environmental Statistical Yearbook of 2014.

### Dependent variable

The dependent variable is the pro-environmental behavior of individuals. In CGSS (2013), there are 10 questions related to pro-environmental behavior including (1) refuse classification, (2) discussion of issues related to environmental protection, (3) bringing own shopping bags, (4) reuse of plastic bags, (5) paying attention to environmental protection issues and information, (6) donation for environmental protection, (7) participation in environmental protection and (8) education activities organized by the government or NGOs, (9) forests maintenance at own expense, and (10) participation in complaints and appeals on environmental protection issues actively. The responses include *never*, *occasionally*, and *frequently*, with assigning values of 1, 2, and 3, respectively. According to Hunter et al. [89], the ten questions are used to compose an index as a proxy of pro-environmental behavior. Firstly, we calculate the average of responses to the 10 questions. Secondly, we use the formula *(X-1) ×100* to obtain the index of pro-environmental behavior ranging from 0 which indicates the lowest level of pro-environmental behavior, to 100 which indicates the highest level of pro-environmental behavior.

### Independent variable

The independent variable is the frequency of using the internet. In CGSS (2013), the corresponding question asks individuals the frequency of using the internet (including mobile phone). The responses include *never*, *rarely*, *sometimes*, *often*, *and very often*, with assigning values ranging from 1 to 5, respectively.

### Control variables

According to existing literature [90–92], we use three kinds of control variables including individual-level control variables, provincial-level control variables and environmental control variables. Individual-level control variables include gender, education level, health conditions, political status, nationality, marital status, age, square of age, and income. The individual-level control variables are all from CGSS (2013) and the response for rejecting, not knowing and not applicable are treated as missing values. Specifically, for gender, Male equals to 1 and Female equals to 0; for education level, from illiteracy to graduate or above are coded from 1 to 14 respectively; for health conditions, unhealthy, relatively healthy, general, relatively healthy and very healthy are coded from 1 to 5 respectively; for political status, the Communist Party members equal to 1 and others equals to 0; for age, it equals 2012 minus the birth year and square of age is also used; for income, it is winsorized at upper and lower 1% and taken logarithm; the fixed-effect dummies for marital status and nationality are also controlled.

The provincial-level control variables are all from National Statistical Yearbook including urbanization rate and GDP per capita of the province. The urbanization rate and GDP per capita are both numerical variables and the GDP per capita is taken logarithm. The environmental control variables are all from National Statistical Yearbook and Environmental Yearbook including the pollution-controlling investment, sulfide emission, NOx emission, and soot emission. They are all numerical variables and taken logarithm.

The descriptive statistics of the pro-environmental behavior are shown in Panel A of the Table 1 and descriptive statistics of all the variables are shown in Panel B of Table 1. The correlations of the variables are shown in Table 2. In empirical analysis, the continuous variables are winsorized at upper and lower 1% to alleviate the influence of extreme values.

**Table 1:** Descriptive statistics.

**Panel A descriptive statistics of the pro-environmental behavior.**					
**Variables**	**Obs**	**Mean**	**S.d**	**Min**	**Max**
Refuse Classification	9,752	1.566	0.702	1	3
Discussion Related Issues	9,752	1.570	0.635	1	3
Bringing Shopping Bags	9,752	2.164	0.787	1	3
Reuse of Plastic Bags	9,752	2.315	0.769	1	3
Donation	9,752	1.186	0.433	1	3
Focus on Related News	9,752	1.635	0.705	1	3
Publicity Campaign	9,752	1.261	0.522	1	3
Environmental Activities	9,752	1.184	0.443	1	3
Forests Maintenance	9,752	1.183	0.476	1	3
Participation in Complaints	9,752	1.101	0.344	1	3
**Panel B descriptive statistics of the variables.**					
**Variables**	**Obs**	**Mean**	**S.d**	**Min**	**Max**
Pro-Environmental Behavior	9,752	25.830	16.600	0	100
Internet Use	9,752	2.178	1.545	1	5
Gender	9,752	1.487	0.500	1	2
Education	9,752	4.908	3.029	1	14
Nationality	9,752	1.390	1.436	1	8
Health Conditions	9,752	3.725	1.077	1	5
Political Status	9,752	3.635	0.940	1	4
Marital Status	9,752	3.216	1.344	1	7
Age	9,752	48.940	16.070	17	84
Income	9,752	22804.390	25796.640	1256	150000
GDP per Capita	9,752	50586.460	21905.700	22862.040	97609.440
Urbanization Rate	9,752	0.575	0.141	0.378	0.896
Polluting Control Investment	9,752	1.462	0.591	0.570	3.010
Sulfide Emission	9,752	70.250	39.770	8.700	164.500
NOx Emission	9,752	77.910	43.390	13.230	165.300
Soot Emission	9,752	41.870	26.500	5.930	131.300

**Table 2:** Spearman correlations of variables.

	Behavior	Internet	Gender	Edu	Health	Political	Nation	Marriage	Age	Income	Urbanrate	GDP	PCI	SOx	NOx	Soot
Behavior	1															
Internet	0.320***	1														
Gender	0.000	-0.060***	1													
Edu	0.413***	0.632***	-0.145***	1												
Health	0.138***	0.317***	-0.060***	0.280***	1											
Political	0.161***	0.114***	-0.156***	0.251***	0.027***	1										
Nation	-0.029***	-0.051***	-0.001	-0.063***	-0.009	-0.003	1									
Marriage	-0.125***	-0.350***	0.112***	-0.315***	-0.202***	-0.004	-0.012	1								
Age	-0.156***	-0.615***	-0.019*	-0.463***	-0.393***	0.076***	-0.037***	0.476***	1							
Income	0.353***	0.510***	-0.205***	0.574***	0.264***	0.199***	-0.091***	-0.151***	-0.305***	1						
Urbanrate	0.259***	0.229***	-0.008	0.273***	0.077***	0.044***	-0.171***	-0.064***	-0.016	0.419***	1					
GDP	0.267***	0.234***	0.011	0.265***	0.105***	0.033***	-0.170***	-0.061***	-0.022**	0.416***	0.932***	1				
PCI	-0.065***	-0.076***	-0.015	-0.066***	0.018*	-0.018*	0.111***	0.014	-0.022**	-0.091***	-0.131***	-0.202***	1			
SOx	-0.134***	-0.097***	0.037***	-0.145***	0.051***	-0.011	-0.071***	0.028***	-0.005	-0.212***	-0.412***	-0.275***	0.007	1		
NOx	-0.139***	-0.079***	0.039***	-0.120***	0.072***	-0.013	-0.142***	0.023**	0.022**	-0.152***	-0.212***	-0.110***	0.028***	0.837***	1	
Soot	-0.182***	-0.139***	0.026***	-0.149***	0.029***	-0.011	-0.052***	0.039***	0.028***	-0.238***	-0.255***	-0.224***	0.247***	0.735***	0.865***	1

Notes:

*** p<0.01

** p<0.05

* p<0.1.

### Regression model

The dependent variable is the index of pro-environmental behavior, a continuous variable. Therefore, we set up linear regression model for the analysis. The baseline model is as follows:

$$Y_{ij}=\alpha_{0}+\alpha_{1}{\mathrm{Internet}}_{ij}+\alpha_{2}X_{ij}+\varepsilon_{ij}$$

where *Y*_*ij*_ is the individual *i*’s pro-environmental behavior at province *j*. *Internet*_*ij*_ is the frequency of using the internet for individual *i* at province *j*. *X*_*ij*_ is a vector of control variables.

## Results

### Internet use and pro-environmental behavior

Table 3 reports the result of the direct relationship between internet use and pro-environmental behavior. Column 1 shows that, without any control variables, the coefficient of internet use is significantly positive, indicating that using the internet makes people behave more environmental friendly. From Column 2 to Column 4, the provincial-level, individual-level and environment-pollution control variables are added respectively, and the coefficients of internet use remain significantly positive, indicating the result is robust with all levels of control variables. Therefore, we conclude that using the internet significantly improves the individual’s pro-environmental behavior. Hypothesis 1 is supported.

**Table 3:** Effects of internet use on pro-environmental behavior.

	(1)	(2)	(3)	(4)
Internet Use	3.283***	2.554***	0.903***	0.859***
	(0.108)	(0.110)	(0.164)	(0.164)
GDP per Capita		-3.564***	-3.040***	-3.473***
		(1.014)	(1.042)	(1.268)
Urbanization Rate		39.823***	30.393***	29.427***
		(3.021)	(3.128)	(4.268)
Gender			2.476***	2.430***
			(0.330)	(0.329)
Education			1.318***	1.313***
			(0.080)	(0.080)
Health			0.477***	0.505***
			(0.163)	(0.163)
Political Status			3.571***	3.609***
			(0.593)	(0.595)
Age			0.309***	0.311***
			(0.070)	(0.070)
Age^2^			-0.002***	-0.002***
			(0.001)	(0.001)
LnIncome			1.520***	1.484***
			(0.176)	(0.176)
PCI				0.290
				(0.282)
Sulfide Emission				2.663***
				(0.708)
NOx Emission				0.575
				(0.837)
Soot Emission				-3.515***
				(0.591)
Constant	18.674***	35.637***	3.910	8.123
	(0.267)	(9.328)	(9.748)	(11.025)
Marriage Fixed-effect	NO	NO	YES	YES
Nationality Fixed-effect	NO	NO	YES	YES
F-Statistics	931.802	586.512	121.673	103.562
Adj_R^2^	0.093	0.155	0.230	0.234
Obs.	9752	9752	8665	8665

Notes: (1) Robust standard errors in parentheses; (2) *** p<0.01, ** p<0.05, * p<0.1. (3) For the questions about Nationality, the response includes: Han, Mongolian, Manchu, Hui, Tibetan, Zhuang, Uighurs and others. A set of dummy variables are used to control the Marriage fixed-effect and Nationality fixed-effect to alleviate the influence of marriage statues and different nationalities.

### Private versus public pro-environmental behavior

We apply factor analysis on the 10 relevant questions in CGSS (2013) to generate measures of private and public pro-environmental behaviors. The results in Table 4 show that questions 1 to 4 and question 6 are loaded on the same factor (private pro-environmental behavior), while the other five questions are loaded on the other factor (public pro-environmental behavior).

**Table 4:** Results of factor analysis.

Variable	Factor 1 Private Pro-Environmental Behavior	Factor 2 Public Pro-Environmental Behavior	Uniqueness
Refuse Classification	**0.510**	0.329	0.632
Discussion of Issues	**0.545**	0.398	0.544
Bring Shopping Bags	**0.753**	0.028	0.432
Reuse of Plastic Bags	**0.739**	-0.045	0.452
Donation	0.160	**0.628**	0.580
Focus on News	**0.473**	0.464	0.561
Publicity Activities	0.216	**0.749**	0.392
Environmental Activities	0.102	**0.796**	0.355
Maintain Forest	-0.128	**0.600**	0.623
Appeals for Issues	-0.064	**0.679**	0.535

The regression results are shown in Table 5. In Columns (1) and (2), without control variables, the coefficients of internet use are both significantly positive, indicating that using the internet helps to increase both private and public pro-environmental behaviors. The results are consistent in Columns (3) and (4) when controlling for the province-level variables, in Columns (5) and (6) when adding individual-level control variables, and in Columns (7) and (8) when adding environment-pollution control variables. Lastly, Seemingly Uncorrelated Regression (SUR) is used to test the whether the coefficients of the internet use are significantly different between the private and public pro-environmental behavior. The p-value is shown in the bottom of Table 5. When there are no control variables, the coefficients of internet use are significantly different between the private and public pro-environmental behavior. When adding the province-level control variables, the coefficients of internet use are still significantly different. Comparing the coefficients of private and public pro-environmental behaviors in Columns (1) and (2) and Columns (3) and (4), we find that internet use has more pronounced impact on private pro-environmental behavior. To be specific, using the internet increases the index of the private and public pro-environmental behaviors by 3.161 and 1.948, respectively, shown in Columns (3) and (4). However, when adding the individual-level control variables, the coefficients of internet use are not significantly different between the private and public pro-environmental behaviors. Therefore, Hypothesis 2 is partially supported.

**Table 5:** Effects of internet use on private and public pro-environmental behaviors.

	(1) Private	(2) Public	(3) Private	(4) Public	(5) Private	(6) Public	(7) Private	(8) Public
Internet Use	4.243*** (0.150)	2.323*** (0.110)	3.161*** (0.152)	1.948*** (0.113)	1.017*** (0.227)	0.789*** (0.180)	1.008*** (0.227)	0.710*** (0.180)
GDP per Capita			45.44*** (4.349)	34.20*** (2.998)	0.168 (1.550)	-6.247*** (0.992)	1.765 (1.803)	-8.710*** (1.339)
Urbanization Rate			-0.306 (1.499)	-6.822*** (0.935)	32.142*** (4.523)	28.645*** (3.166)	27.052*** (6.063)	31.802*** (4.466)
Gender					5.092*** (0.474)	-0.140 (0.339)	5.083*** (0.473)	-0.223 (0.338)
Education					1.839***	0.797***	1.831***	0.794***
					(0.108)	(0.089)	(0.108)	(0.089)
Health					0.552**	0.401**	0.574**	0.437***
					(0.240)	(0.159)	(0.240)	(0.159)
Political Status					3.742***	3.401***	3.775***	3.443***
					(0.785)	(0.656)	(0.786)	(0.659)
Age					0.320***	0.297***	0.330***	0.291***
					(0.100)	(0.072)	(0.100)	(0.073)
Age^2^					-0.002**	-0.003***	-0.002**	-0.003***
					(0.001)	(0.001)	(0.001)	(0.001)
LnIncome					2.360***	0.680***	2.350***	0.619***
					(0.258)	0.789***	(0.259)	(0.171)
PCI							1.808***	-1.228***
							(0.426)	(0.280)
Sulfide Emission							4.880***	0.446
							(1.062)	(0.663)
NOx Emission							-1.389	2.539***
							(1.236)	(0.834)
Soot Emission							-4.269***	-2.761***
							(0.866)	(0.565)
Constant	33.251***	4.098***	12.75	58.53***	-32.319**	40.139***	-48.423***	64.670***
	(0.400)	(0.410)	(13.81)	(8.548)	(14.481)	(9.200)	(15.857)	(11.281)
Marriage Fixed-effect	NO	NO	NO	NO	YES	YES	YES	YES
Nationality Fixed-effect	NO	NO	NO	NO	YES	YES	YES	YES
F-Statistics	802.683	443.555	580.084	206.227	119.694	44.727	102.412	39.709
Adj_R2	0.077	0.052	0.142	0.074	0.215	0.110	0.220	0.116
Obs.	9752	9752	9752	9752	8665	8665	8665	8665
P-value	0.000		0.000		0.354		0.224	

Notes: (1) Robust standard errors in parentheses; (2) *** p<0.01, ** p<0.05, * p<0.1; (3) Seeming Uncorrelated Regression (SUR) is used to test whether the coefficients of the internet use are significantly different between the private and public pro-environmental behavior. The null hypothesis is that the coefficients of the internet use for private and public pro-environmental behavior are equal. (4) For the questions about Nationality, the response includes: Han, Mongolian, Manchu, Hui, Tibetan, Zhuang, Uighurs and others. A set of dummy variables are used to control the Marriage fixed-effect and Nationality fixed-effect to alleviate the influence of marriage statues and different nationalities.

### The heterogeneity of pro-environmental behavior

In order to identify the heterogeneous effects of internet use on pro-environmental behavior, we further investigate the effects across groups of different income, gender and location. Panel A of Table 6 shows the heterogeneous effects across income groups. We divide the whole sample by income level into three groups: low-income (the bottom one-third of the sample), middle-income (the middle one-third of the sample) and high-income (the top one-third of the sample). Results show that internet use increases the index of pro-environment behavior by 1.442, 0.846 and 0.924 for low-income, middle-income and high-income groups, respectively. The Fisher’s permutation test is used to test whether the coefficients of internet use are significantly different between each group. The empirical p-value shows that the influence of internet use on pro-environmental behavior between low-income and middle-income groups, and between low-income and high-income groups are both significantly different at the 10% level. However, the influence between middle-income and high-income groups is not statistically significantly different. The results partially support Hypothesis 3a.

**Table 6:** Heterogeneity effects of internet use on pro-environmental behavior.

Dependent Variable: Pro-Environmental Behavior				
**Panel A**				
**Subgroup: Income**		**Low-Income**	**Middle-Income**	**High-Income**
	Internet Use	1.442***	0.846***	0.924***
		(0.486)	(0.275)	(0.241)
	Control Variables	YES	YES	YES
	Marriage Fixed-effect	YES	YES	YES
	Nationality Fixed-effect	YES	YES	YES
	F-Statistics	9.821	23.153	31.379
	Adj_R^2^	0.089	0.162	0.179
	Obs	2234	3240	3191
Empirical p-value	Low-income versus Middle-income	0.100		
	Low-income versus High-income	0.100		
	Middle-income versus High-income	0.391		
**Panel B**				
**Subgroup: Gender**		**Male**	**Female**	
	Internet Use	0.529**	1.331***	
		(0.224)	(0.243)	
	Control Variables	YES	YES	
	Marriage Fixed-effect	YES	YES	
	Nationality Fixed-effect	YES	YES	
	F-Statistics	54.963	58.948	
	Adj_R^2^	0.209	0.273	
	Obs	4742	3923	
Empirical p-value	Male versus Female	0.005		
**Panel C**				
**Subgroup: Location**		**Urban**	**Rural**	
	Internet Use	0.700***	0.678**	
		(0.196)	(0.321)	
	Control Variables	YES	YES	
	Marriage Fixed-effect	YES	YES	
	Nationality Fixed-effect	YES	YES	
	F-Statistics	57.789	88.461	
	Adj_R^2^	0.203	0.092	
	Obs	5377	3288	
Empirical p-value	Urban versus Rural	0.466		

Notes: (1) Robust standard errors in parentheses; (2) *** p<0.01, ** p<0.05, * p<0.1; (3) Control variables include individual-level, provincial-level and environment-pollution variables; (4) Fisher’s permutation test is used to test whether the coefficients of the internet use are significantly different between each group; empirical p-value is used to test the coefficients difference between each group, which is calculated by bootstrapping 1000 times; the null hypothesis is that the coefficients of internet use for each group are equal. (5) For the questions about Nationality, the response includes: Han, Mongolian, Manchu, Hui, Tibetan, Zhuang, Uighurs and others. A set of dummy variables are used to control the Marriage fixed-effect and Nationality fixed-effect to alleviate the influence of marriage statues and different nationalities.

Panel B of Table 6 shows the results comparing female and male sub-samples. In general, internet use has positive influence on pro-environmental behavior for both gender groups. However, using the internet significantly increases the index of pro-environment behavior by 1.331 for females and 0.529 for males. The results of the Fisher’s permutation test show that the coefficients of internet use for females and males are significantly different, indicating the effect is more pronounced for females, supporting Hypothesis 3b. Panel C of Table 6 shows the results comparing urban and rural residents. Specifically, internet use increases the index of pro-environmental behavior by 0.700 and 0.678 for individuals located in urban and rural areas, respectively. The Fisher’s permutation test also shows that the coefficients of internet use for urban and rural residents are not statistically significantly different, not supporting Hypothesis 3c.

Extended from Tables 6 and 7 shows the differential heterogeneous effects between private and public pro-environment behaviors. Panel A of Table 7 shows the results across income groups. Specifically, internet use increases the index of private pro-environmental behavior by 1.950, 1.172 and 1.264 for the low-income, middle-income and high-income groups, respectively. The paralleling numbers for the index of public pro-environmental behavior are 0.935, 0.520 and 0.583. SUR is applied to test whether the coefficients of the private and public pro-environmental behavior are significantly different. The results of SUR show that the coefficients of internet use for private and public pro-environmental behavior are significantly different between the middle and high-income groups. However, within the low-income group, the coefficients are not significantly different between private and public pro-environmental behavior. Furthermore, the Fisher’s permutation test is used to test whether the coefficients for each group are significantly different. The results show that the private pro-environmental behavior is significantly different for low-income versus middle-income groups, and between low-income versus high-income groups, both at the 10% significance level. Both sets of results point to the conclusion that internet use has the most pronounced influence for the low-income group and even stronger effect on the private pro-environmental behavior compared to the public type.

**Table 7:** Heterogeneity effects of internet use on private and public pro-environmental behaviors.

Dependent Variable: Pro-Environmental Behavior							
**Panel A**							
**Subgroup: Income**		**Low-Income**		**Middle-Income**		**High-Income**	
		**Private**	**Public**	**Private**	**Public**	**Private**	**Public**
	Internet Use	1.950***	0.935*	1.172***	0.520*	1.264***	0.583**
		(0.691)	(0.489)	(0.387)	(0.291)	(0.324)	(0.277)
	Control Variables	YES	YES	YES	YES	YES	YES
	Marriage Fixed-effect	YES	YES	YES	YES	YES	YES
	Nationality Fixed-effect	YES	YES	YES	YES	YES	YES
	F-Statistics	7.948	7.837	21.642	10.960	34.187	13.189
	Adj_R^2^	0.063	0.085	0.141	0.093	0.202	0.071
	Obs	2234	2234	3240	3240	3191	3191
	P-value (Private versus Public)	0.144		0.100		0.059	
	Empirical p-value	Low-Income versus Middle-Income		Low-Income versus High-Income		Middle-Income versus High-Income	
	**Private**	0.100		0.100		0.467	
	**Public**	0.211		0.240		0.406	
**Panel B**							
**Subgroup: Gender**		**Male**		**Female**			
		**Private**	**Public**	**Private**	**Public**		
	Internet Use	0.620**	0.439*	1.603***	1.058***		
		(0.311)	(0.246)	(0.330)	(0.270)		
	Control Variables	YES	YES	YES	YES		
	Marriage Fixed-effect	YES	YES	YES	YES		
	Nationality Fixed-effect	YES	YES	YES	YES		
	F-Statistics	55.276	21.743	55.846	21.770		
	Adj_R^2^	0.199	0.100	0.249	0.135		
	Obs	4742	4742	3923	3923		
	P-value (Private versus Public)	0.593		0.100			
	Empirical p-value	Male versus Female					
	**Private**	0.018					
	**Public**	0.045					
**Panel C**							
**Subgroup: Location**		**Urban**		**Rural**			
		**Private**	**Public**	**Private**	**Public**		
	Internet Use	0.886***	0.514**	0.625	0.730**		
		(0.265)	(0.224)	(0.458)	(0.326)		
	Control Variables	YES	YES	YES	YES		
	Marriage Fixed-effect	YES	YES	YES	YES		
	Nationality Fixed-effect	YES	YES	YES	YES		
	F-Statistics	53.271	27.799	111.883	99.343		
	Adj_R^2^	0.193	0.101	0.063	0.078		
	Obs	5377	5377	3288	3288		
	P-value (Private versus Public)	0.821		0.205			
	Empirical p-value	Urban versus Rural					
	**Private**	0.301					
	**Public**	0.288					

Notes: (1) Robust standard errors in parentheses; (2) *** p<0.01, ** p<0.05, * p<0.1; (3) Control variables include individual-level, provincial-level and environment-pollution variables; (4) Seeming Uncorrelated Regression (SUR) is used to test whether the coefficients of the internet use are significantly different between the private and public pro-environmental behavior. The null hypothesis is that the coefficients of the internet use for private and public pro-environmental behavior are equal; (5) Fisher’s permutation test is used to test whether the coefficients of the internet use are significantly different between each group; empirical p-value is used to test the coefficients difference between each group, which is calculated by bootstrapping 1000 times; the null hypothesis is that the coefficients of the internet use for each group are equal. (6) For the questions about Nationality, the response includes: Han, Mongolian, Manchu, Hui, Tibetan, Zhuang, Uighurs and others. A set of dummy variables are used to control the Marriage fixed-effect and Nationality fixed-effect to alleviate the influence of marriage statues and different nationalities.

Panel B of Table 7 shows the results between female and male groups. Internet use increases the index of private pro-environmental behavior by 1.603 and 0.620 for females and males, respectively. The paralleling numbers for the public ones are 1.058 and 0.439. The results of SUR show that the coefficients of private and public pro-environmental behavior within females and males are significantly different. The Fisher’s permutation test shows that the coefficients of the private and public pro-environmental behavior between females and males are significantly different. Both sets of results suggest that internet use has stronger effect among females than males, and the difference is even more pronounced on the private pro-environmental behavior. Panel C of Table 7 shows the results between urban and rural residents. The internet use increases the index of the private and public pro-environmental behavior by 0.886 and 0.514, respectively, for urban residents, while for rural residents internet use only significantly improves the index of the public pro-environmental behavior by 0.730. The results of SUR and the Fisher’s permutation test show that neither the coefficients of private and public pro-environmental behavior within the urban and rural residents nor those between the two groups are significantly different.

### Robustness checks

We perform several robustness checks on the results. Firstly, we use internet leisure as an alternative measure of internet use. Secondly, we use ordered-logit model to test the relationship between internet use and pro-environmental behavior. Thirdly, we apply the instrumental variable approach in order to alleviate the endogeneity problem.

#### Internet leisure and pro-environmental behavior

CGSS (2013) includes a question inquiring the respondents whether they surf the internet at their spare time. The responses include *every day*, *several times a week*, *several times a month*, *several times a year or less*, and *never*. The variable is coded 5–1, respectively, to describe the frequency of using the internet. We add control variables gradually and the results are shown in Table 8. We find that when gradually adding control variables, the coefficients of the internet leisure is significantly positive, which indicates that the results are robust.

**Table 8:** Robustness check 1: Internet leisure.

	(1)	(2)	(3)	(4)
Internet Leisure	3.107***	2.446***	1.070***	1.022***
	(0.099)	(0.101)	(0.147)	(0.147)
Provincial Level	NO	YES	YES	YES
Individual Level	NO	NO	YES	YES
Environment-Pollution	NO	NO	NO	YES
Marriage Fixed-effect	NO	NO	YES	YES
Nationality Fixed-effect	NO	NO	YES	YES
F-Statistics	991.613	610.184	123.098	104.677
Adj_R^2^	0.098	0.159	0.232	0.236
Obs.	9736	9736	8652	8652

Notes: (1) Robust standard errors in parentheses; (2) *** p<0.01, ** p<0.05, * p<0.1; (3) Control variables include individual-level, provincial-level and environment-pollution variables. (4) For the questions about Nationality, the response includes: Han, Mongolian, Manchu, Hui, Tibetan, Zhuang, Uighurs and others. A set of dummy variables are used to control the Marriage fixed-effect and Nationality fixed-effect to alleviate the influence of marriage statues and different nationalities.

#### Ordered-logit model

In the main regressions, we use an aggregated index to measure the pro-environmental behavior. In the robustness check, we apply ordered-logit model to each of the ten questions regarding pro-environmental behavior. The results are shown in Table 9. Among the ten pro-environment behaviors, seven are statistically significantly affected by internet use, suggesting robust results.

**Table 9:** Robustness check 2: Ordered-logit model.

	Refuse Classification	Discussion of Issues	Bringing Shopping Bags	Reuse of Plastic Bags	Donation
Internet Use	0.048**	0.094***	0.023	0.001	0.080***
	(0.021)	(0.022)	(0.021)	(0.021)	(0.027)
Control Variables	YES	YES	YES	YES	YES
Marriage Fixed-effect	YES	YES	YES	YES	YES
Nationality Fixed-effect	YES	YES	YES	YES	YES
Log-likelihood	-7654.491	-7169.583	-8822.031	-8629.305	-4065.283
Pseudo Adj_R^2^	0.072	0.097	0.054	0.024	0.092
Obs.	8665	8665	8665	8665	8665
	**Focus on News**	**Publicity Campaigns**	**Environmental Activities**	**Maintain Forest**	**Appeals for** **Issues**
Internet Use	0.123***	0.114***	0.048*	0.016	0.086**
	(0.022)	(0.026)	(0.028)	(0.029)	(0.036)
Control Variables	YES	YES	YES	YES	YES
Marriage Fixed-effect	YES	YES	YES	YES	YES
Nationality Fixed-effect	YES	YES	YES	YES	YES
Log-likelihood	-7777.124	-5041.848	-4123.884	-4245.040	-2774.269
Pseudo Adj_R^2^	0.093	0.104	0.086	0.028	0.059
Obs.	8665	8665	8665	8665	8665

Notes: (1) Robust standard errors in parentheses; (2) *** p<0.01, ** p<0.05, * p<0.1; (3) Control variables include individual-level, provincial-level and environment-pollution variables. (4) For the questions about Nationality, the response includes: Han, Mongolian, Manchu, Hui, Tibetan, Zhuang, Uighurs and others. A set of dummy variables are used to control the Marriage fixed-effect and Nationality fixed-effect to alleviate the influence of marriage statues and different nationalities.

#### Province and city fixed-effects

In the main regressions, we use a set of co-variates to control for the effect of economic factors. However, some unobserved heterogeneity at the province and city levels may have influence on the environmental behaviors. Therefore, we control for the province and city fixed-effects in robustness check and the results are shown in Table 10. The coefficient of internet use is still significantly positive, indicating robustness of the results.

**Table 10:** Robustness check 3: Province and city fixed-effect.

	(1)	(2)
Internet Use	0.811***	0.762***
	(0.161)	(0.158)
Individual Level	YES	YES
Marriage Fixed-effect	YES	YES
Nationality Fixed-effect	YES	YES
Province Fixed-effect	YES	NO
City Fixed-effect	NO	YES
F-Statistics	84.32	34.03
Adj_R^2^	0.268	0.330
Obs.	8665	8665

Notes: (1) Robust standard errors in parentheses; (2) *** p<0.01, ** p<0.05, * p<0.1; (3) Control variables include individual-level, provincial-level and environment-pollution variables. (4) For the questions about Nationality, the response includes: Han, Mongolian, Manchu, Hui, Tibetan, Zhuang, Uighurs and others. A set of dummy variables are used to control the Marriage fixed-effect and Nationality fixed-effect to alleviate the influence of marriage statues and different nationalities.

#### Whether using the internet or not

In the main regression, we treat the response of the internet use from 1 to 5, which may overestimate the results for those who never use the internet. In robustness check, the response of never is treated as 0 and others are treated as 1. The results are shown in Table 11, which indicates the results are robust.

**Table 11:** Robustness check 4: Whether using the internet or not.

	(1)	(2)	(3)	(4)
Internet Use (0/1)	10.398***	8.322***	3.913***	3.878***
	(0.330)	(0.332)	(0.462)	(0.463)
Provincial Level	NO	YES	YES	YES
Individual Level	NO	NO	YES	YES
Environment-Pollution	NO	NO	NO	YES
Marriage Fixed-effect	NO	NO	YES	YES
Nationality Fixed-effect	NO	NO	YES	YES
F-Statistics	9752	9752	8665	8665
Adj_R^2^	0.096	0.160	0.229	0.234
Obs.	994.902	629.389	218.385	165.719

Notes: (1) Robust standard errors in parentheses; (2) *** p<0.01, ** p<0.05, * p<0.1; (3) Control variables include individual-level, provincial-level and environment-pollution variables. (4) For the questions about Nationality, the response includes: Han, Mongolian, Manchu, Hui, Tibetan, Zhuang, Uighurs and others. A set of dummy variables are used to control the Marriage fixed-effect and Nationality fixed-effect to alleviate the influence of marriage statues and different nationalities.

#### Instrumental variables

We use two instrumental variables to alleviate the potential endogeneity issues. The first one is the number of cell-phone base stations at each province. More cell-phone base stations indicate a region with higher level of infrastructure related to the internet; therefore, people can use the internet more frequently and conveniently. However, it is irrelevant with people’s behavior, because building such infrastructure is not determined by individuals. The second instrumental variable is the number of web pages at each province. More web pages indicate a region with more developed internet industry and people are more likely to use the internet; however, it is not relevant with people’s pro-environmental behavior. The results of the 2SLS model are shown in Table 12. Panel A shows the result with the number of base stations as the instrumental variable, and Panel B shows the result with the number of web pages as the instrumental variable. The corresponding coefficients of internet use in all models are significantly positive, suggesting robustness after alleviating the possible endogenous bias.

**Table 12:** Robustness check 5: Instrumental variables.

**Panel A**			
	**(1)**	**(2)**	**(3)**
Base Stations	11.950*** (0.633)	17.304*** (1.882)	16.238*** (3.750)
Provincial Level	NO	YES	YES
Individual Level	NO	YES	YES
Environment-Pollution	NO	NO	YES
Marriage Fixed-effect	NO	YES	YES
Nationality Fixed-effect	NO	YES	YES
Under identification Test	438.901	133.402	35.344
Weak Identification Test	570.214	156.203	37.348
**Panel B**			
	**(1)**	**(2)**	**(3)**
Web Pages	12.648*** (0.720)	20.211*** (2.315)	18.341*** (3.875)
Provincial Level	NO	YES	YES
Individual Level	NO	YES	YES
Environment-Pollution	NO	NO	YES
Marriage Fixed-effect	NO	YES	YES
Nationality Fixed-effect	NO	YES	YES
Underidentification Test	365.662	107.386	33.931
Weak Identification Test	487.478	119.913	39.057

Notes: (1) Robust standard errors in parentheses; (2) *** p<0.01, ** p<0.05, * p<0.1; (3) Control variables include individual-level, provincial-level and environment-pollution variables; (4) LM statistic is used to test under identification; Wald F statistic is used to test weak identification. (5) For the questions about Nationality, the response includes: Han, Mongolian, Manchu, Hui, Tibetan, Zhuang, Uighurs and others. A set of dummy variables are used to control the Marriage fixed-effect and Nationality fixed-effect to alleviate the influence of marriage statues and different nationalities.

### Plausible channels

The main results have supported that the use of internet significantly increases pro-environmental behaviors. We further discuss the plausible channels through which internet use affects pro-environmental behavior, including providing information, encouraging participation in pro-environmental campaigns and improving social relationships. Firstly, the channel of providing information is verified to see whether internet use increases the environmental awareness and environment-protection knowledge. Secondly, the channel of encouraging participating in pro-environmental campaigns is verified to see whether using the internet encourages the individuals to participate in more campaigns. Lastly, another channel of social relationships is explored to see whether internet use improves social relationships. Specifically, Question B21 and Question B25 in CGSS (2013) are used to measure environmental awareness and environment-protection knowledge; Question D25 and the number of letters and visits on environmental issues in each province is used to measure the channel of encouraging participating in pro-environmental campaigns; and Question A31a and Question B5 are used to measure social relationships. In order to test the plausible channels, the dependent variables are replaced by the variables mentioned above. The results reported in Table 13 show that the coefficients of internet use are all significantly positive for the plausible channels, which indicates that internet use may affect pro-environmental behavior through providing information, encouraging participation in pro-environmental campaigns and improving social relationships.

**Table 13:** Plausible channels.

	Information		Campaigns		Social Relationship	
	Environmental Awareness	Environmental Knowledge	Impacts	Supervisions	Contacts	Social
Internet Use	0.108***	0.058*	0.080***	0.022***	0.023***	0.025**
	(0.031)	(0.032)	(0.016)	(0.007)	(0.009)	(0.011)
Control Variables	YES	YES	YES	YES	YES	YES
Marriage Fixed-effect	YES	YES	YES	YES	YES	YES
Nationality Fixed-effect	YES	YES	YES	YES	YES	YES
F-Statistics	65.519	5.389	9.357	214.255	19.684	10.533
Adj_R^2^	0.152	0.080	0.062	0.354	0.056	0.030
Obs.	8658	1004	2189	8665	8659	8662

Notes: (1) Robust standard errors in parentheses; (2) *** p<0.01, ** p<0.05, * p<0.1; (3) Control variables include individual-level, provincial-level and environment-pollution variables; (4) For *Environmental Awareness* and *Environmental Knowledge*, in CGSS (2013), Question B22 and Question B25 include a series of questions asking whether the respondents know about a variety of environmental issues or the knowledge of environmental protection. We compose the variables measuring environmental awareness and environmental knowledge by counting the number of questions to which the answer is yes; (5) for *Impacts*, in CGSS (2013), Question D15 asks the respondents whether the information (texts, pictures and videos) obtained from the internet affect the thoughts and behavior; the variable *Impacts* is coded as 1 to 4 for the responses of no impacts, fewer impacts, some impacts and great impacts, respectively; for *Supervisions*, the logarithm of (1+the number of letters and visits on environmental issues in each province) is used; (6) For *Contacts*, Question B5 asks how often the respondents contact with the relatives and friends; the response is coded from 1 to 5 for rarely close to very close, respectively; for *Social*, Question A31a asks whether the respondents would like to have some social activities during their spare time; the response are coded from 1 to 5 for never to frequently, respectively.

## Discussion

In recent decades, applying different variables, methods and dataset, scholars have explored external and individual factors that promote the pro-environmental behavior [4, 5], such as information, knowledge, education, social relations, social participation and motivation [7–9]. In fact, all these factors are closely related to the rapid development of the internet, that is, the widely spread use of internet may play an effective role in promoting pro-environmental behavior. However, the function of the internet on pro-environment behavior is neglected to some extent due to the data limitation. China has become one of the countries with the greatest number of internet users and urgent demand of solving current environmental problems, providing us the opportunity to study the relationship between internet use and pro-environmental behavior, using the representative survey data of CGSS (2013).

Different from almost of the existing literature, this paper proposes a theoretical framework based on TPB theory to support and explain the relationship between internet use and pro-environmental behavior. In addition, some determinants of pro-environmental behavior neglected by prior research, such as types of pro-environmental behaviors, the heterogeneity of impacts, and plausible channels, have been studied in detail. Our findings imply that internet use positively influences the individuals’ pro-environmental behavior, which is regarded as an empirical evidence for the theoretical framework we proposed. We further divide the pro-environmental behavior into private and public types. The result shows that both of these two types of behavior are affected by internet use and the private type receives relatively more pronounced influence compared with the public type. When considering the heterogeneity across different groups of individuals, another novel finding is that the positive effect of internet use on pro-environmental behavior is more pronounced among low-income and female groups, compared to middle to high income and male counterparts, respectively.

Compared with previous studies, the main contributions of the present study are twofold. First, we extend the stream of research (e.g., [80, 93–96]) on the factors affecting pro-environmental behavior by providing a comprehensive analytic framework which is embedded in the digital era. Second, from the perspective of the internet, we supplement the research about the function of the internet on the individuals’ behavior [97, 98]. Under the circumstance of rapid development of the internet, the results support the positive influence of internet use on individuals’ behavior. Therefore, combining the extant literature and the results we obtain, we can offer a better and deeper understanding of the factors affecting the pro-environmental behavior and the function of the internet.

## Conclusion

The factors promoting the pro-environmental behavior are critical to resolve the urgent environmental tensions. Based on CGSS 2013 data, this study focuses on exploring the effect of individuals’ internet use on their pro-environmental behaviors from the perspectives of the types of pro-environmental behaviors, the heterogeneity of impacts, and plausible channels. Based on the empirical findings, we propose that developing the internet technology and the industry can achieve both economy growth and the improvement of environmental quality. The role of the internet, such as providing the environmental information, popularizing environmental knowledge, optimizing individual or household energy use decisions and improving the social relationships, can all contribute to improving pro-environmental behavior. Therefore, several effective measures should be adopted to strengthen the positive effects of the internet on pro-environmental behavior. For example, more applications about the environment, such as recording carbon footprint and inquiring the environmental information of the commodities, can be developed to provide people more chances to behave environmental- friendly. In addition, more pro-environmental campaigns can be organized or NGOs on environment can be established in order to enhance the positive effects of internet use on public pro-environmental behavior.

However, there are still several limitations. Firstly, due to data limitations, only CGSS (2013) cross-sectional data set is employed. Due to the repaid development of the internet, especially the mobile internet, the results of the internet use on pro-environment behavior may be underestimated. Secondly, we cannot further distinguish specific kinds of internet use such as using the internet for work or leisure, or using the internet for searching the information or for relaxing. Using the internet for different purposes or under different occasions may affect the pro-environmental behavior differently. Therefore, future research may want to focus on the differential effect across situations. Thirdly, for the plausible channels, we only provide some preliminary results due to data limitation. In the future, the channels should be tested more carefully. Last but not the least, the result we get is short-term influence. Future research may want to find the long-term relationship between internet use and pro-environmental behavior when panel data are available.
